# Supplementary material for: A fault-intrusion-tolerant system and deadline-aware algorithm for scheduling scientific workflow in the cloud
Source: PeerJ Comput Sci. 2021 Nov 2;7:e747. doi: 10.7717/peerj-cs.747 (PMC8576554; doi:10.7717/peerj-cs.747)
Supplement: Supplemental Information 6 [file peerj-cs-07-747-s006.zip › Myproject1.1/docs/index-files/index-4.html]

D-Index


JavaScript is disabled on your browser.


- Overview
- Package
- Class
- Use
- Tree
- Deprecated
- Index
- Help

- Prev Letter
- Next Letter

- Frames
- No Frames

- All Classes

A B C D E F G H I J L M N O P Q R S T U V W Z 


## D

DataAwareScheduler - Class in org.workflowsim.scheduler
:   Data aware algorithm.

DataAwareScheduler() - Constructor for class org.workflowsim.scheduler.DataAwareScheduler


Datacenter - Class in org.cloudbus.cloudsim
:   Datacenter class is a CloudResource whose hostList are virtualized.

Datacenter(String, DatacenterCharacteristics, VmAllocationPolicy, List<Storage>, double) - Constructor for class org.cloudbus.cloudsim.Datacenter
:   Allocates a new PowerDatacenter object.

DatacenterBroker - Class in org.cloudbus.cloudsim
:   DatacentreBroker represents a broker acting on behalf of a user.

DatacenterBroker(String) - Constructor for class org.cloudbus.cloudsim.DatacenterBroker
:   Created a new DatacenterBroker object.

DatacenterCharacteristics - Class in org.cloudbus.cloudsim
:   DatacenterCharacteristics represents static properties of a resource such as resource
    architecture, Operating System (OS), management policy (time- or space-shared), cost and time
    zone at which the resource is located along resource configuration.

DatacenterCharacteristics(String, String, String, List<? extends Host>, double, double, double, double, double) - Constructor for class org.cloudbus.cloudsim.DatacenterCharacteristics
:   Allocates a new DatacenterCharacteristics object.

datacenterCharacteristicsList - Variable in class org.cloudbus.cloudsim.DatacenterBroker
:   The datacenter characteristics list.

DatacenterExtended - Class in org.workflowsim
:   DatacenterExtended extends Datacenter so as we can use CondorVM and other
    components

DatacenterExtended(String, DatacenterCharacteristics, VmAllocationPolicy, List<Storage>, double) - Constructor for class org.workflowsim.DatacenterExtended


datacenterid - Variable in class org.cloudbus.cloudsim.network.datacenter.Switch


datacenterIdsList - Variable in class org.cloudbus.cloudsim.DatacenterBroker
:   The datacenter ids list.

datacenterRequestedIdsList - Variable in class org.cloudbus.cloudsim.DatacenterBroker
:   The datacenter requested ids list.

DATAcloudlet\_SUBMIT - Static variable in class org.cloudbus.cloudsim.DataCloudTags
:   Denotes the submission of a DataCloudlet.

DataCloudTags - Class in org.cloudbus.cloudsim
:   This class contains additional tags for the DataCloud functionalities, such as file information
    retrieval, file transfers, and storage info.

dataTransferTime(List<File>, Cloudlet, int) - Method in class org.workflowsim.scheduler.DataAwareScheduler


dc - Variable in class org.cloudbus.cloudsim.network.datacenter.Switch


deadline - Variable in class org.cloudbus.cloudsim.network.datacenter.AppCloudlet


deallocateBwForAllVms() - Method in class org.cloudbus.cloudsim.provisioners.BwProvisioner
:   Releases BW used by a all VMs.

deallocateBwForAllVms() - Method in class org.cloudbus.cloudsim.provisioners.BwProvisionerSimple


deallocateBwForVm(Vm) - Method in class org.cloudbus.cloudsim.provisioners.BwProvisioner
:   Releases BW used by a VM.

deallocateBwForVm(Vm) - Method in class org.cloudbus.cloudsim.provisioners.BwProvisionerSimple


deallocateHostForVm(Vm) - Method in class org.cloudbus.cloudsim.network.datacenter.NetworkVmAllocationPolicy
:   Releases the host used by a VM.

deallocateHostForVm(Vm) - Method in class org.cloudbus.cloudsim.power.PowerVmAllocationPolicyAbstract


deallocateHostForVm(Vm) - Method in class org.cloudbus.cloudsim.VmAllocationPolicy
:   Releases the host used by a VM.

deallocateHostForVm(Vm) - Method in class org.cloudbus.cloudsim.VmAllocationPolicySimple
:   Releases the host used by a VM.

deallocateMipsForAllVms() - Method in class org.cloudbus.cloudsim.provisioners.PeProvisioner
:   Releases MIPS used by all VMs.

deallocateMipsForAllVms() - Method in class org.cloudbus.cloudsim.provisioners.PeProvisionerSimple


deallocateMipsForVm(Vm) - Method in class org.cloudbus.cloudsim.provisioners.PeProvisioner
:   Releases MIPS used by a VM.

deallocateMipsForVm(Vm) - Method in class org.cloudbus.cloudsim.provisioners.PeProvisionerSimple


deallocatePesForAllVms() - Method in class org.cloudbus.cloudsim.VmScheduler
:   Releases PEs allocated to all the VMs.

deallocatePesForAllVms() - Method in class org.cloudbus.cloudsim.VmSchedulerTimeShared
:   Releases PEs allocated to all the VMs.

deallocatePesForVm(Vm) - Method in class org.cloudbus.cloudsim.Host
:   Releases PEs allocated to a VM.

deallocatePesForVm(Vm) - Method in class org.cloudbus.cloudsim.VmScheduler
:   Releases PEs allocated to a VM.

deallocatePesForVm(Vm) - Method in class org.cloudbus.cloudsim.VmSchedulerSpaceShared


deallocatePesForVm(Vm) - Method in class org.cloudbus.cloudsim.VmSchedulerTimeShared


deallocateRamForAllVms() - Method in class org.cloudbus.cloudsim.provisioners.RamProvisioner
:   Releases BW used by a all VMs.

deallocateRamForAllVms() - Method in class org.cloudbus.cloudsim.provisioners.RamProvisionerSimple


deallocateRamForVm(Vm) - Method in class org.cloudbus.cloudsim.provisioners.RamProvisioner
:   Releases BW used by a VM.

deallocateRamForVm(Vm) - Method in class org.cloudbus.cloudsim.provisioners.RamProvisionerSimple


DEFAULT\_BAUD\_RATE - Static variable in class org.cloudbus.cloudsim.core.CloudSimTags
:   Denotes the default baud rate for some CloudSim entities

DEFAULT\_MTU - Static variable in class org.cloudbus.cloudsim.DataCloudTags
:   Default Maximum Transmission Unit (MTU) of a link in bytes

DEFAULT\_STORAGE\_SIZE - Static variable in class org.cloudbus.cloudsim.DataCloudTags
:   The default storage size (10 GByte)

deferred - Static variable in class org.cloudbus.cloudsim.core.CloudSim
:   The deferred event queue.

DeferredQueue - Class in org.cloudbus.cloudsim.core
:   This class implements the deferred event queue used by `Simulation`.

DeferredQueue() - Constructor for class org.cloudbus.cloudsim.core.DeferredQueue


delayLength - Variable in class org.workflowsim.failure.FailureRecord
:   delay length.

delayMatrix - Static variable in class org.cloudbus.cloudsim.NetworkTopology


DelayMatrix\_Float - Class in org.cloudbus.cloudsim.network
:   This class represents an delay-topology storing every distance between connected nodes

DelayMatrix\_Float(TopologicalGraph, boolean) - Constructor for class org.cloudbus.cloudsim.network.DelayMatrix\_Float
:   this constructor creates an correct initialized Float-Delay-Matrix

deleteFile(String) - Method in class org.cloudbus.cloudsim.HarddriveStorage
:   Removes a file from the storage.

deleteFile(String, File) - Method in class org.cloudbus.cloudsim.HarddriveStorage
:   Removes a file from the storage.

deleteFile(File) - Method in class org.cloudbus.cloudsim.HarddriveStorage
:   Removes a file from the storage.

deleteFile(String, File) - Method in class org.cloudbus.cloudsim.SanStorage
:   Removes a file from the storage.

deleteFile(File) - Method in class org.cloudbus.cloudsim.SanStorage
:   Removes a file from the storage.

deleteFile(String) - Method in interface org.cloudbus.cloudsim.Storage
:   Removes a file from the storage.

deleteFile(String, File) - Method in interface org.cloudbus.cloudsim.Storage
:   Removes a file from the storage.

deleteFile(File) - Method in interface org.cloudbus.cloudsim.Storage
:   Removes a file from the storage.

deleteRequiredFile(String) - Method in class org.cloudbus.cloudsim.Cloudlet
:   Deletes the given filename from the list.

depth - Variable in class org.workflowsim.failure.FailureRecord
:   the depth (also used as type in some cases) of a failure.

disable() - Static method in class org.cloudbus.cloudsim.Log
:   Disables the output.

DistanceVariance - Class in org.workflowsim.clustering.balancing.metrics
:   A DistanceVariance

DistanceVariance() - Constructor for class org.workflowsim.clustering.balancing.metrics.DistanceVariance


downlinkbandwidth - Variable in class org.cloudbus.cloudsim.network.datacenter.Switch


downlinkswitches - Variable in class org.cloudbus.cloudsim.network.datacenter.Switch


downlinkswitchpktlist - Variable in class org.cloudbus.cloudsim.network.datacenter.Switch


Dvfs - Class in org.cloudbus.cloudsim.examples.power.planetlab
:   A simulation of a heterogeneous power aware data center that only applied DVFS, but no dynamic
    optimization of the VM allocation.

Dvfs() - Constructor for class org.cloudbus.cloudsim.examples.power.planetlab.Dvfs


Dvfs - Class in org.cloudbus.cloudsim.examples.power.random
:   A simulation of a heterogeneous power aware data center that only applied DVFS, but no dynamic
    optimization of the VM allocation.

Dvfs() - Constructor for class org.cloudbus.cloudsim.examples.power.random.Dvfs

A B C D E F G H I J L M N O P Q R S T U V W Z

- Overview
- Package
- Class
- Use
- Tree
- Deprecated
- Index
- Help

- Prev Letter
- Next Letter

- Frames
- No Frames

- All Classes
